# Nutrition before and during Surgery and the Inflammatory Response of the Heart: A Randomized Controlled Trial

**DOI:** 10.1155/2015/123158

**Published:** 2015-07-29

**Authors:** Marlieke Visser, Hans W. M. Niessen, Wouter E. M. Kok, Riccardo Cocchieri, Willem Wisselink, Paul A. M. van Leeuwen, Bas A. J. M. de Mol

**Affiliations:** ^1^Department of Cardiothoracic Surgery, Academic Medical Center, University of Amsterdam, P.O. Box 22700, 1100 DE Amsterdam, Netherlands; ^2^Department of Surgery, VU University Medical Center, P.O. Box 7057, 1007 MB Amsterdam, Netherlands; ^3^Department of Pathology and Cardiac Surgery, ICaR-VU, VU University Medical Center, P.O. Box 7057, 1007 MB Amsterdam, Netherlands; ^4^Department of Cardiology, Academic Medical Center, University of Amsterdam, P.O. Box 22700, 1100 DE Amsterdam, Netherlands

## Abstract

Major surgery induces a long fasting time and provokes an inflammatory response which increases the risk of infections. Nutrition given before and during surgery can avoid fasting and has been shown to increase the arginine/asymmetric dimetlhylarginine ratio, a marker of nitric oxide availability, in cardiac tissue and increased concentrations of branched chain amino acids in blood plasma. However, the effect of this new nutritional strategy on organ inflammatory response is unknown. Therefore, we studied the effect of nutrition before and during cardiac surgery on myocardial inflammatory response. In this trial, 32 patients were randomised between enteral, parenteral, and no nutrition supplementation (control) from 2 days before, during, up to 2 days after coronary artery bypass grafting. Both solutions included proteins or amino acids, glucose, vitamins, and minerals. Myocardial atrial tissue was sampled before and after revascularization and was analysed immunohistochemically, subdivided into cardiomyocytic, fatty, and fibrotic areas. Inflammatory cells, especially leukocytes, were present in cardiac tissue in all study groups. No significant differences were found in the myocardial inflammatory response between the enteral, parenteral, and control groups. In conclusion, nutrition given before and during surgery neither stimulates nor diminishes the myocardial inflammatory response in patients undergoing coronary artery bypass grafting. The trial was registered in Netherlands Trial Register (NTR): NTR2183.

## 1. Introduction

Surgery provokes an inflammatory response in order to heal tissue damage but the same inflammatory response predisposes patients to the development of infections [1]. Surgical patients commonly receive only clear fluids during the period prior to surgery and the day after surgery leading to starvation of the patient over a longer period of time. Fasting can induce thirst, stress, insulin resistance [2], and nutrient deficiencies which can impair immune defence [3]. As most surgical patients are already in a catabolic state [4], prolonged fasting will impair recovery after surgery [2]. A previous study of patients undergoing cardiac surgery found that preoperative nutritional supplements decreased plasma concentrations of interleukin 6 (IL-6) and reduced the number of postoperative infections [5]. In other surgical populations too, pre- and postoperative nutritional supplementation has been found to reduce the number of postoperative infections [6]. Recently, for the first time, nutrition was given before, during, and after cardiac surgery in order to avoid a long fasting time [7]. It was found that this new nutritional strategy increased the arginine/asymmetric dimethylarginine (ADMA) ratio, a marker of nitric oxide (NO) availability, in cardiac tissue. Furthermore, nutrition increased the arginine/ADMA ratio and concentrations of branched chain amino acids (BCAA, i.e., leucine, isoleucine, and valine) in blood plasma, and an increase in plasma arginine/ADMA ratio correlated with improved myocardial viability. Both NO [8] and amino acids [9] are known to play an essential role in the inflammatory response. NO enhances immunity at a cellular level by increasing proliferation of lymphocytes and monocytes, enhancing T-helper cell formation, activating macrophage cytotoxicity, reinforcing natural killer cells, increasing phagocytosis, and enhancing cytokine production [8].

The amino acid arginine is an immune enhancing nutrient and plays an important role in immunity by many pathways from which its roles as substrate of the enzymes arginase and nitric oxide synthase (NOS) are most familiar [8]. However, while arginine is essential in the inflammatory response, high levels of the amino acids can result in negative outcome [10, 11]. Here, arginine might have induced excessive NO production by inducible nitric oxide synthase (iNOS) which in turn could have led to detrimental systemic vasodilation [8]. On the other hand, arginine is needed as precursor of NO mediated microvascular vasodilatation facilitated by endothelial NOS (eNOS) which is crucial for organ perfusion and coronary patency. Likely, NO availability needs to be perfectly balanced [12]. Therefore, nutritional formulas must be used that contain arginine but not in elevated levels. Furthermore, inadequate access to amino acids during immune cell activation may diminish immune response by inhibiting immune cell division, differentiation, and migration [9]. Therefore, by increasing myocardial and plasma arginine/ADMA ratio and amino acids, supplementation of nutrition before and during surgery without elevated concentrations of the immune enhancing nutrients might be a safe way that may influence myocardial inflammatory response.

Patients undergoing coronary artery bypass grafting (CABG) offer the possibility to study the effects of nutrition on the inflammatory response of the heart as cardiac tissue biopsies can be taken during this procedure. In order to prevent cardioplegic effects on cardiac cells, patients that can undergo an off-pump (i.e., without cardiopulmonary bypass) CABG procedure should be selected. Therefore, in this proof-of-concept trial, we investigated the effect of nutrition before and during surgery on the myocardial inflammatory response in patients undergoing off-pump CABG.

## 2. Materials and Methods

### 2.1. Study Design

This randomised controlled intervention study was carried out at the Department of Cardiothoracic Surgery at the Academic Medical Center of the University of Amsterdam (AMC) between July 2010 and August 2012. The study protocol [14] was approved by the Medical Ethics Committee of the AMC and the competent authority in Netherlands. A monitor verified that the trial was carried out in accordance with the protocol as described in the European Medicine Agency's “Note for guidance on good clinical practice CPMP/ICH/135/95” as well as the Declaration of Helsinki. Monitoring was performed and reported following the sponsor's standing operating procedures.

The primary end points of the study were cardiomyocytes structure (i.e., immunohistochemical analysis) and the concentrations of amino acids in myocardial tissue.

### 2.2. Nutrition Protocol

Patients were randomised to the enteral (*n* = 12), parenteral (*n* = 9), or control (*n* = 11) group. Randomization was performed online via a secure internet facility in a 1 : 1 : 1 ratio by the TENALEA Clinical Trial Data Management System using randomly permuted blocks of sizes three and six.

#### 2.2.1. Enteral Nutrition

During the four days before hospital admission, the enteral group received 125 mL per day of a nutrient drink (Nutridrink Compact, Nutricia, Zoetermeer, Netherlands) consisting of proteins, carbohydrates, fats, vitamins, and minerals (Table 1). When admitted to the hospital, patients in the enteral group received a solution containing amino acids (PeptoPro, DSM, Delft, Netherlands), carbohydrates (Fantomalt, Nutricia, Zoetermeer, Netherlands), and vitamins and minerals (Phlexy-Vits, SHS International Ltd., Liverpool, UK) which was prepared at the hospital each day. An amount of 1050 mL of the enteral nutrition was given during 24 hours. This nutrition was given two days before, during, and two days after CABG by a computerized guidance system-placed nasoduodenal tube (Cortrak, Viasys Healthcare, Wheeling, IL, USA). On the morning of surgery, the position of the duodenal tube was verified. Patients were permitted to eat and drink in addition to their supplemental nutrition.

#### 2.2.2. Parenteral Nutrition

Patients in the parenteral group received 1250 mL of nutrition (Nutriflex Lipid peri, B. Braun, Oss, Netherlands) containing amino acids, lipids, and glucose. An amount of 1250 mL of the amino acid infusion (840 mOsm/L) was given in 24 hours for 5 days (Table 3). In addition, vitamins (Cernevit, Baxter, Utrecht, Netherlands) and trace elements (Nutritrace, B. Braun, Oss, Netherlands) were added to the parenteral nutrition. This nutrition was given two days before, during, and two days after CABG. Patients were permitted to eat and drink in addition to their supplemental nutrition.

#### 2.2.3. Controls

The control group followed the standard protocol of the department of cardiothoracic surgery of the AMC allowing patients to eat and drink until six hours before surgery. The day after surgery, this standard protocol prescribes a (clear) liquid diet. On the second day after surgery, a normal diet is recommended for patients.

### 2.3. Patients

All 32 patients were due to undergo off-pump cardiac surgery for CABG and were aged between 55 and 76 years. Exclusion criteria were a combined valve and CABG procedure, pregnancy, renal insufficiency (defined as creatinine >95 *μ*mol/L for women and >110 *μ*mol/L for men), and liver insufficiency (defined as alanine aminotransferase >34 U/L for women and >45 U/L for men). All patients gave informed consent.

### 2.4. Surgical Procedures

Standard anesthesthetic and off-pump CABG surgical procedures were used in this study.

### 2.5. Myocardial Tissue Sampling

During surgery, two tissue samples of the appendix of the right atrium were taken by the surgeon. One sample was taken after the harvesting of the left mammary artery and before performing the distal anastomosis and one sample at the end of the procedure before closing the pericardium. Each sample was immediately put in formalin prior to immunohistochemical analysis.

### 2.6. Immunohistochemical Analyses

In myocardial samples, inflammatory cells were analysed and quantified using antibodies against CD45 (lymphocytes), myeloperoxidase (MPO: neutrophilic granulocytes), and CD68 (macrophages). In addition, cytokines (interleukin-6 (IL-6), IL-1*β*, and tumour necrosis factor-*α* (TNF-*α*)) were determined in endothelial cells. Endothelial cell activation was also analysed using antibodies against complement (C3d), P-selectin, E-selectin, and the advanced glycation end product (AGE) carboxymethyl lysine (CML). Complement was further analysed in erythrocytes and fibrotic tissue.

Tissue samples fixed in formalin were embedded in paraffin and sliced in 4 *μ*m sections. Slides were deparaffinised and hydrated, and endogenous peroxidase activity was blocked by 0.03% hydrogen peroxide in methanol for 30 minutes. Enzymatic CD68, CML, E-selectin, and P-selectin antigen retrieval was done by incubating the tissue samples with 0.1% pepsin (activated with hydrochloric acid 37%, 1 : 600) for 30 minutes at 37°C. MPO, CD45, and C3d heat antigen retrieval was performed by heating the slides for 15 minutes in citrate (MPO; pH 6.0) and in Tris-EDTA (CD45; pH 9.0) at 100°C. After washing the sections in demineralised water and in PBS (pH 7.4), the slides were incubated with specific antibody solutions (diluted in PBS-BSA) for 60 minutes (anti-CD68 1 : 100, anti-MPO 1 : 500, anti-CD45 1 : 50, anti-CML 1 : 500, anti-C3d 1 : 1000, anti-E-selectin, and anti-P-selectin 1 : 50). Enzymatic IL-6, IL-1*β*, and TNF-*α* antigen retrieval was performed by boiling slides in a citrate pH 6.0 (IL-6 and IL-1*β*) or a Tris-EDTA pH 9.0 (TNF-*α*) solution in a microwave for 10 minutes. Next, sections were incubated with 1 : 500 monoclonal rabbit anti-human IL-1*β* (Abcam, UK) and 1 : 1000 monoclonal mouse anti-human TNF-*α* (Abcam, UK) for 1 hour at room temperature. For IL-6 staining, sections were incubated overnight with 1 : 100 monoclonal rabbit anti-human IL-6 (Santa Cruz, USA) at room temperature.

The slides were again washed in PBS, followed by 30-minute incubation with anti-rabbit and anti-mouse EnVision-HRP (DakoCytomation, Denmark). Staining was visualised using 3,3-diaminobenzidine (DAB, 0.1 mg/mL, 0,02% H_2_O_2_). Sections were counterstained with haematoxylin, dehydrated, and covered. The same staining procedures were used as a control, but instead of the primary monoclonal or polyclonal antibody, PBS was used; these heart tissue slides were found to be negative.

### 2.7. Morphometrical Analyses

In atrial tissue, fatty tissue and fibrotic areas are found next to cardiomyocytic areas in amounts that vary not only between patients but also within different areas of the atrium of one patient. In all these three areas of the atria (as seen by histological view) within each myocardial tissue slide, the number of CD45-, CD68-, MPO-, and IL-6-positive inflammatory cells and E-selectin-, P-selectin-, IL-1*β*-, TNF-*α*-, and C3d-positive endothelial vessels was measured separately as the number of positive cells or vessels/mm^2^ myocardium using Q-PRODIT (Leica, Cambridge, UK). Due to the high number of CD45-positive cells in fibrotic tissue, separate positive cells could not be identified and quantified as such. Therefore, the surface of the CD45-positive area was calculated and presented per *μ*m^2^ myocardium. Additionally, C3d-positive erythrocytes and fibrotic areas were graded based on an intensity score; namely, 1 = no or weak intensity, 2 = moderate intensity, and 3 = strong intensity. CML-positive endothelial cells were semiquantified based on an intensity score for each positive vessel as follows: 1 = weak positivity, 2 = moderate positivity, and 3 = strong positivity [15]. Each CML intensity score was multiplied by the number of vessels that scored positively. The multiplication scores were then added and the sum was divided by the area of the slide, resulting in an immunohistochemical score per mm^2^.

### 2.8. Statistical Analysis

Data were expressed as mean ± standard deviation (SD) where data were normally distributed and as median with interquartile range (IQR) where data were not normally distributed. To investigate the effects of administration of enteral or parenteral nutrition during surgery, differences between the study groups in the first and second tissue samples were analysed using the Kruskal-Wallis test. A *p* value of < 0.05 (2-tailed) was considered statistically significant. All statistical analyses were performed with SPSS 20.0 for Windows (SPSS Inc., Chicago, IL, USA).

## 3. Results and Discussion

### 3.1. Patient Characteristics and Myocardial Tissue Samples

Although 38 patients were enrolled, only 32 patients were ultimately included. In two patients, the CABG procedure was switched from off-pump to on-pump (which was an exclusion criterion). In one patient, the appendix of the right atrium could not be reached, the atrial tissue of one patient was lost during immunohistochemical analysis, and in two patients the peripheral line was removed before surgery. Patient, laboratory and operation characteristics, and data about postoperative outcome are presented in Table 2. Concerning these characteristics, no statistically significant differences between the groups were observed.

### 3.2. The Effect of Nutrition during Surgery on Myocardial Inflammatory Response

Table 3 shows the number of inflammatory cells and cytokines and endothelial cell activation in the first and second myocardial tissue samples in each study group. Mean time between sampling of the first and second tissue samples was 97 ± 28 min.

The first tissue samples comprised (median (IQR)) 56.6% (27.8–74.7) cardiomyocyte areas, 29.5% (22.7–45.0) fibrotic areas, and 0.98% (0.00–26.3) fatty areas. In the second tissue samples, these values were 56.5% (14.0–73.6), 31.1% (15.6–42.6), and 0.82% (0.00–33.30), respectively. These percentages had no statistically significant differences between the first and second tissue samples.

Lymphocytes were present in all three areas in both the first and the second tissue samples. Neutrophilic granulocytes in cardiomyocytic and fibrotic tissue were present in the second tissue sample but were not present in most samples of all areas taken at the start of surgery and of fibrotic and fatty tissue taken at the end of surgery. Macrophages were not present in most tissue samples of all three areas, both at the start and at the end of surgery. IL-6 was frequently present in cardiomyocytic areas of both the first and the second tissue samples. In fibrotic and fatty tissue areas, IL-6 was not found in most samples taken at the start and the end of surgery. IL-1*β* was not shown in most endothelial cells in all areas of the atria of the first and second tissue samples. TNF-*α* was not found in endothelial vessels of the first or second tissue samples. In all areas of the atria, P-selectin was present in both the first and the second tissue samples while E-selectin was not found in the first tissue sample and in very low numbers in the second tissue sample. CML was present in both the first and the second tissue samples of all areas of the atria. C3d was not present in most endothelium of all areas of the atria of the first and second tissue samples. C3d-positive erythrocytes and fibrotic areas were found in both the first and the second tissue samples.

In both the first and the second tissue samples, the number of inflammatory cells and cytokines and endothelial cell activation did not differ significantly between the enteral, parenteral, and control groups.

### 3.3. Discussion

In this study, the effect of nutritional supplementation before and during surgery on the myocardial inflammatory response in cardiac surgery was investigated. While inflammatory cells were demonstrated in cardiac tissue at the start and end of surgery, nutrition before and during cardiac surgery did not affect myocardial inflammatory response in these patients.

Both enteral and parenteral nutrition included amino acids. Amino acids are essential for a proper immune cell response [9]. When immune cells are activated by inflammatory signals, their demand for amino acids increases rapidly which may explain the low plasma levels of amino acids in surgical patients [16]. In a previous analysis of this study data, nutrition before and during surgery increased the myocardial and plasma arginine/ADMA ratio which may have beneficial effects on myocardial glucose metabolism [7]. Furthermore, plasma concentrations of BCAA were higher in the enteral and parenteral groups than in the control group. Increasing BCAA might be beneficial as they function as precursors for myocardial protein synthesis [17, 18] while inflammation is known to increase protein degradation and to attenuate protein synthesis [19]. By being precursor of NO, the amino acid arginine is important for regulation of inflammation and immunity [8]. However, production of NO can be reduced by the NO synthase (NOS) inhibitor ADMA which is considered a risk factor for cardiovascular disease and an indicator of worse outcome in patients with cardiac dysfunction [20, 21]. The net production of NO probably depends on the ratio between substrate and inhibitor: the arginine/ADMA ratio. While previous data analysis showed that nutrition before and during surgery increased the arginine/ADMA ratio and BCAA [7], nutrition during surgery did not affect myocardial inflammatory response.

A lack of any effect of our nutritional intervention may be found in the relatively low amount of immune-modulating nutrients and low amount of calories in the (par)enteral nutrition. However, as this was the first study investigating the effect of nutrition during surgery, we supplied hypocaloric nutrition which included the immunomodulator arginine but not in elevated concentrations [14]. These characteristics of our nutrition are supported by results from recent studies showing that high levels of immunomodulating nutrients [22, 23] and overfeeding [24–27] can have negative effects on clinical outcome. The negative effect of the immunomodulating nutrients is ascribed to their stimulating effects on proliferation of immune cells [28] and production of excessive NO [21] and thereby augmenting the inflammatory response which negatively influences outcome. In inflammatory states, supplementation of high amounts of arginine or glutamine may induce an excess in NO production by iNOS which can be deleterious because it may lead to detrimental vasodilatation [8] and to increased formation of ROS leading to cellular damage [29]. Furthermore, glutamine might stimulate lymphocyte proliferation and cytokine production and may enhance the immune response [28]. Therefore, immunomodulating nutrition is not recommended in inflammatory states [21, 28, 30]. On the other hand, an increase in NO facilitated by eNOS is of vital importance as it mediates microvascular vasodilatation. Likely, NO availability needs to be perfectly balanced [12, 21]. The negative findings of overfeeding in previous studies are ascribed to the increase in fat mass and hyperglycemia [24–27]. For example, early parenteral nutrition in addition to enteral nutrition has been shown to induce fat incorporation in muscle tissue and to negatively affect clinical outcome [24, 25]. Results of previous studies investigating the effect of hypocaloric nutrition show contrary results. In the ICU, hypocaloric nutrition has been shown to have similar [31] and negative effects [32] compared to normocaloric nutrition. However, both studies included patients requiring artificial nutrition who might not have received enough protein and/or calories while in our study patients received nutrition in order to avoid fasting. Additionally, we hypothesize that a lack of physical activity plays an important role in the lack of any (beneficial) effect in our study and previous studies as nutritional interventions may be most effective together with anabolic stimuli [33–37]. Probably, nutritional supplementation with an anabolic stimulus like physical activity may result in an increase in muscle mass and strength (instead of fat mass) which is related to better clinical outcome [38, 39]. Unfortunately, studies combining the effects of nutrition and physical activity are very scarce. Therefore, future studies investigating nutritional supplementation should incorporate an anabolic stimulus, like exercise training, in their study protocol.

As a first in line, this study had limitations. First, it was a proof-of-concept trial that tested the novel strategy of nutrition during surgery as a form of perioperative treatment. The sample size was powered for the primary outcome (i.e., amino acids and immunohistochemistry in myocardial tissue) and a significant difference between the (par)enteral and control groups. Unfortunately, the intended sample size could not be reached because of the low consent numbers. Nevertheless, in the current study population, our results show that it is feasible to supply nutrition during surgery which did not affect myocardial inflammation. No adverse events were seen in our study. Second, it is not known if the inflammatory cells are evenly distributed throughout the right atrial appendix and other parts of the myocardium. However, the availability of small samples of atrial appendix obtained in the course of off-pump CABG provided a sound model to study inflammatory response effects. Finally, true baseline values of the inflammatory response in myocardial tissue could not be measured as it was not possible to take myocardial biopsies before surgery.

## 4. Conclusions

Our study demonstrated the presence of inflammatory cells in the heart of patients undergoing off-pump CABG. Nutrition before and during surgery neither stimulated nor diminished the inflammatory response of the myocardium. Future studies should focus on the effects of nutritional supplementation in combination with physical activity and investigate whether continuing of nutritional interventions during surgery is beneficial for immune response and clinical outcome.

## Figures and Tables

**Table 1:** Composition of enteral and parenteral nutrition.

	Enteral group		Parenteral group
	Drink (at home) per day	Nutrition (at hospital) per day	Nutrition (at hospital) per day
Volume (mL)	125	1050	1250
Amino acids (g)	12	80.5	40
Carbohydrates (g)	37.1	95	80
Fat (g)	11.6	1.5	50
Energy (kcal)	300	745	955
Vitamins and minerals	Yes	Yes	Yes

**Table 2:** Patient characteristics and postoperative outcome.

	Enteral group (*n* = 12)	Parenteral group (*n* = 9)	Control group (*n* = 11)
Patients			
Age (years)	66.1 ± 6.1	66.6 ± 7.1	63.3 ± 6.7
Gender (% male)	12 (100)	10 (100)	11 (100)
BMI (kg/m^2^)	27.8 (27.0–30.9)	27.4 (26.2–30.3)	27.7 (24.2–30.0)
Fat-free mass index (kg/m^2^)	19.8 (18.3–21.0)	19.5 (18.2–21.1)	20.9 (18.4–21.7)
Diabetes mellitus (%)	3 (25.0)	4 (44.4)	3 (27.3)
EuroSCORE^*∗*^	2.0 (1.3–3.0)	3.0 (3.0–4.0)	2.0 (0.0–3.0)
Previous acute myocardial infarction (%)	5 (41.7)	6 (66.7)	2 (18.2)
Preoperative laboratory tests			
Plasma CRP (mg/L)	0.8 (0.7–1.6)	2.7 (0.9–5.9)	0.6 (0.6–3.2)
Plasma albumin (g/L)	46.5 (45.3–48.8)	43.0 (41.0–47.0)	46.0 (45.0–48.0)
Plasma NT-proBNP (ng/L)	234 (54–522)	204 (121–439)	99 (71–431)
Intraoperative period			
Propofol use (%)	8 (66.7)	4 (44.4)	8 (72.7)
Surgery duration (min)	268 ± 39	279 ± 27	294 ± 40
Postoperative period			
Plasma CK-MB (*µ*g/L)	9.0 (6.4–15.0)	8.2 (6.2–8.9)	10.3 (7.6–15.5)
Intensive care stay (hours)	21.5 (18.0–23.8)	24.0 (22.0–34.5)	21.0 (20.0–22.0)
Stent (%)	0 (0)	0 (0)	0 (0)
Catheterisation < 3 months (%)	0 (0)	0 (0)	1 (9.1)
Revascularisation < 3 months (%)	0 (0)	0 (0)	0 (0)
Infections < 3 months (%)	0 (0)	0 (0)	1 (9.1)
Mortality (%)	0 (0)	0 (0)	0 (0)

Values are expressed as median (IQR) or mean ± SD. ^*∗*^EuroSCORE, European System for Cardiac Operation Risk Evaluation score. The EuroSCORE is a validated risk stratification system to determine the risk profile for mortality of cardiothoracic surgery patients [13].

**Table 3:** Inflammatory cells in the 1st and 2nd myocardial tissue samples (number/mm^2^) in study groups.

	Start of surgery (1st tissue sample)			End of surgery (2nd tissue sample)		
	Enteral group (*n* = 12)	Parenteral group (*n* = 9)	Control group (*n* = 11)	Enteral group (*n* = 12)	Parenteral group (*n* = 9)	Control group (*n* = 11)
Lymphocytes (CD45)						
Cardiomyocytic area	5.03 (1.58–15.17)	0.00 (0.00–8.64)	10.23 (3.44–15.51)	5.75 (0.81–6.88)	3.92 (0.00–6.53)	1.91 (0.00–10.10)
Fibrotic area (*µ*m^2^)	154.10 (35.43–1215.60)	93.90 (36.17–2080.91)	241.18 (120.80–481.83)	178.09 (31.01–5449.95)	47.39 (9.23–147.22)	60.25 (0.08–138.74)
Fatty area	8.54 (0.89–12.54)	4.06 (0.16–10.43)	6.22 (0.00–120.05)	3.22 (0.00–5.08)	2.56 (1.19–93.43)	7.24 (3.22–22.85)
Neutrophil granulocytes (MPO)						
Cardiomyocytic area	0.00 (0.00–14.23)	0.00 (0.00–0.31)	1.47 (0.00–3.62)	4.10 (0.00–15.86)	3.07 (0.32–6.53)	0.65 (0.00–1.94)
Fibrotic area	0.00 (0.00–0.00)	0.00 (0.00–11.87)	0.00 (0.00–1.72)	0.00 (0.00–32.44)	6.72 (0.00–12.92)	1.64 (0.00–15.56)
Fatty area	0.00 (0.00–0.00)	0.00 (0.00–0.16)	0.00 (0.00–0.00)	0.00 (0.00–1.81)	0.00 (0.00–2.49)	0.00 (0.00–8.52)
Macrophages (CD68)						
Cardiomyocytic area	0.00 (0.00–0.00)	0.00 (0.00–0.00)	0.00 (0.00–1.03)	0.00 (0.00–0.00)	0.00 (0.00–1.12)	0.00 (0.00–0.38)
Fibrotic area	0.00 (0.00–0.95)	0.00 (0.00–9.86)	1.72 (0.00–8.24)	0.00 (0.00–4.32)	0.72 (0.00–13.49)	2.11 (0.00–6.62)
Fatty area	0.00 (0.00–0.49)	0.00 (0.00–1.28)	0.00 (0.00–1.05)	0.00 (0.00–1.21)	0.00 (0.00–2.32)	0.00 (0.00–0.66)
IL-6						
Cardiomyocytic area	28.72 (0.48–77.38)	0.00 (0.00–18.91)	19.20 (1.20–88.94)	36.03 (0.00–80.77)	20.53 (0.79–43.25)	14.53 (0.00–55.17)
Fibrotic area	0.00 (0.00–7.04)	0.00 (0.00–0.000)	2.47 (0.00–6.39)	0.00 (0.00–2.26)	0.00 (0.00–5.90)	0.00 (0.00–3.37)
Fatty area	0.00 (0.00–0.00)	0.00 (0.00–2.50)	0.00 (0.00–0.00)	0.00 (0.00–0.00)	0.00 (0.00–1.67)	0.00 (0.00–0.00)
IL-1*β* endothelium	0.00 (0.00–0.60)	0.00 (0.00–0.50)	0.00 (0.00–1.72)	0.00 (0.00–1.86)	0.30 (0.00–0.79)	0.00 (0.00–0.00)
TNF-*α* endothelium	0.00 (0.00–0.00)	0.00 (0.00–0.00)	0.00 (0.00–0.00)	0.00 (0.00–0.00)	0.00 (0.00–0.00)	0.00 (0.00–0.00)
Activated endothelium						
P-selectin	2.17 (0.17–3.76)	1.41 (0.87–5.43)	3.37 (0.69–6.29)	2.29 (0.57–6.93)	3.33 (1.27–5.06)	1.70 (0.32–6.46)
E-selectin	0.00 (0.00–0.00)	0.00 (0.00–0.30)	0.00 (0.00–0.00)	0.17 (0.00–0.48)	0.14 (0.00–0.46)	0.00 (0.00–0.42)
Proinflammatory vessel damage (CML)	26.00 (7.58–77.44)	20.78 (5.23–68.66)	46.31 (4.12–134.68)	28.78 (5.29–85.01)	21.18 (12.12–45.80)	21.21 (9.28–81.26)
C3d						
Endothelium	0.00 (0.00–0.36)	0.09 (0.00–0.25)	0.00 (0.00–0.36)	0.00 (0.00–0.32)	0.00 (0.00–0.27)	0.00 (0.00–0.14)
Erythrocytes intensity score > 1	41.7%	22.2%	9.1%	58.3%	44.4%	27.3%
Fibrotic area intensity score > 1	8.3%	11.1%	9.1%	75.0%	55.6%	54.5%

Values are median (IQR).

MPO, myeloperoxidase; CML, carboxymethyl lysine.
